# Arrhythmia Classification Based on Multi-Domain Feature Extraction for an ECG Recognition System

**DOI:** 10.3390/s16101744

**Published:** 2016-10-20

**Authors:** Hongqiang Li, Danyang Yuan, Youxi Wang, Dianyin Cui, Lu Cao

**Affiliations:** 1 School of Electronics and Information Engineering, Tianjin Polytechnic University, Tianjin 300387, China; 1431096018@stu.tjpu.edu.cn (D.Y.); wangyouxi@tjpu.edu.cn (Y.W.); 1430092037@stu.tjpu.edu.cn (D.C.); 2 Tianjin Chest Hospital, Tianjin 300222, China; caolutj@yahoo.com.cn

**Keywords:** ECG recognition system, multi-domain features, kernel-independent component analysis, support vector machine

## Abstract

Automatic recognition of arrhythmias is particularly important in the diagnosis of heart diseases. This study presents an electrocardiogram (ECG) recognition system based on multi-domain feature extraction to classify ECG beats. An improved wavelet threshold method for ECG signal pre-processing is applied to remove noise interference. A novel multi-domain feature extraction method is proposed; this method employs kernel-independent component analysis in nonlinear feature extraction and uses discrete wavelet transform to extract frequency domain features. The proposed system utilises a support vector machine classifier optimized with a genetic algorithm to recognize different types of heartbeats. An ECG acquisition experimental platform, in which ECG beats are collected as ECG data for classification, is constructed to demonstrate the effectiveness of the system in ECG beat classification. The presented system, when applied to the MIT-BIH arrhythmia database, achieves a high classification accuracy of 98.8%. Experimental results based on the ECG acquisition experimental platform show that the system obtains a satisfactory classification accuracy of 97.3% and is able to classify ECG beats efficiently for the automatic identification of cardiac arrhythmias.

## 1. Introduction

With the progress of science and technology, automatic analysis and diagnosis systems based on electrocardiogram (ECG) signals have been extensively investigated to detect and diagnose cardiac diseases [1,2,3,4,5]. The use of an ECG automatic analysis and diagnosis system not only reduces the workload of doctors but also improves diagnostic efficiency and accuracy. ECG is a synthesized reflection of heart electrical activity in the human body used to obtain the cardiac rhythm and electrical conduction of physiological and pathological information [6,7]. However, ECG signals are often mixed with various noises and artefacts. As a result, examiners experience difficulty distinguishing normal signals from arrhythmia signals. Pre-processing of ECG signals is necessary to reduce various interferences. Some techniques based on digital filters have been employed to remove noise and power-line interference from biomedical signals [8,9,10]. Other mathematical morphological algorithms are applied in signal pre-processing [11,12]. Wavelet transform (WT) method based on its good time-frequency property for signal denoising have been popularly developed and applied to eliminate noise effectively in ECG identification system [13,14,15,16]. In addition, an adaptive filtering algorithm for ECG denoising was proposed and compared with the denoising method based on wavelet shrinkage [17]. An approach based on empirical mode decomposition and an improved approximate envelope method was also presented for ECG signal processing [18].

Previous studies have demonstrated the successful application of various feature extraction and classification methods. ECG features mainly include time domain, frequency domain, morphological and nonlinear features. Classification methods mainly focus on random forest [19], linear discriminant classification, neural networks (NNs), support vector machine (SVM), etc. As ECG waveforms in time domains are complicated and easily interfered by noise, classic time domain analysis had low accuracy in extracting features [20,21]. Thereafter, the transform and morphological methods are studied to obtain ECG features containing lots of time and frequency information [19,22]. However, these features can not reflect more accurate characteristics of ECG for achieving high classification accuracy. Thus, combining several methods has become popular in ECG feature extraction and classification recently. A wavelet optimization approach based on the combination of the polyphase representation of wavelets, particle swarm optimization for feature extraction and the SVM classifier was employed in the classification of ECG signals [23]. Lin extracted normalized R-R interval and morphological features as the input features to train and test the linear discriminant classifier in the classification of heartbeats [24]. A cross-correlation-based approach was used to extract suitable features, and a least squares SVM (LSSVM) classifier was developed to classify ECG beats [25]. Several independent component analysis (ICA) algorithms were tested and analysed to identify various components with high accuracy in a particular algorithm based on biomedical data for classification [26]. The extracted bispectral features were subjected to principle component analysis (PCA) for dimensionality reduction and then input into a four-layer feed-forward NN and LSSVM for classification [27]. Valenza proposed a personalized probabilistic framework wherein features were derived from instantaneous spectrum and bispectrum; an SVM classifier was applied in heartbeat recognition [28]. In a previous study, multiclass-directed acyclic graph SVM was implemented on feature vectors utilising empirical mode decomposition and singular value decomposition for ECG classification [29]. Kamath studied ECG beats from the energy perspective by extracting features from the nonlinear component in time and frequency domains via the Teager energy operator and used an NN as a classifier to identify the five classes of ECG beats [30].

Although the studies mentioned above can also produced good classification, they only used the combination of time domain, frequency domain and linear features, or used single nonlinear features in ECG classification, which can not represent more complex characteristics of ECG signals. Therefore, the current study proposes an ECG recognition system that extracts multi-domain features through kernel-independent component analysis (KICA) [31] and discrete wavelet transform (DWT). Before feature extraction, ECG signals are pre-processed with an improved threshold method based on wavelet transform for ECG denoising. PCA and linear discriminant analysis (LDA) [32] are applied to reduce feature dimensions. Multi-domain features, which contain abundant characteristic information on ECG, are used as input to train and test an SVM classifier. The SVM classifier is optimized by the genetic algorithm (GA) [33] and applied to categorise ECG beats into five types, namely, normal beat (N), left bundle branch block beat (LBBB), right bundle branch block beat (RBBB), premature ventricular contraction (PVC) and atrial premature beat (APC) [27,34]. The ECG arrhythmia recordings employed in this study are derived from the MIT-BIH arrhythmia database, which is a complete system that began distributing information in 1980 and can be accessed from the PhysioBank. An ECG acquisition experimental platform is also constructed to acquire ECG data from Fluke ProSim^TM^2 vital sign simulator (Fluke Corp., Everett, WA, USA) to demonstrate the effectiveness of the proposed ECG recognition system. The experimental results based on ECG data collected from the experimental platform show that the proposed system can achieve satisfactory identification results.

## 2. Materials and Methods 

### 2.1. The Proposed System Based on the Multi-Domain Feature Extraction

**Figure 1:**
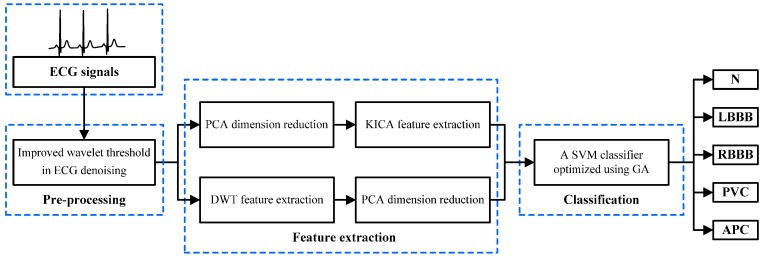
Block scheme of the proposed electrocardiogram (ECG) recognition system for ECG beats classification. The presented system is composed of ECG pre-processing, feature extraction and classification. ECG pre-processing removes noise and interference from original ECG beats. Feature extraction derives multi-domain features through kernel-independent component analysis (KICA) and discrete wavelet transform (DWT). The support vector machine (SVM) classifier, optimized with genetic algorithm (GA), divides ECG beats into five categories: normal beat (N), left bundle branch block beat (LBBB), right bundle branch block beat (RBBB), premature ventricular contraction (PVC) and atrial premature beat (APC).

The entire block diagram of the proposed ECG recognition system for ECG beats classification is shown in Figure 1. An ECG recognition system based on multi-domain feature extraction is proposed to identify cardiac arrhythmias. The proposed system consists of three main parts, namely, ECG pre-processing, feature extraction and classification; its procedure is illustrated by the following steps.

1 The ECG beats are pre-processed to eliminate disturbance and noise by denoising with an improved wavelet threshold method.
2 The original ECG data are optimized through PCA. KICA is applied to reduce data dimensions and obtain the nonlinear features of ECG beats. The DWT method is employed to extract frequency domain features. The maximum, minimum, mean and standard deviation values of each sampling signal wavelet coefficient are calculated. Additionally, LDA is used to optimise the frequency domain features.
3 The multi-domain features are composed of nonlinear and frequency domain features, which are used as input features to train and test the SVM classifier model. GA is employed to optimise the SVM parameters and improve the classifier’s performance. Finally, the five types of ECG beats derived from the MIT-BIH arrhythmia database are classified with the optimised SVM classifier.

### 2.2. ECG Pre-Processing Based on the Improved Wavelet Threshold Method

**Figure 2:**
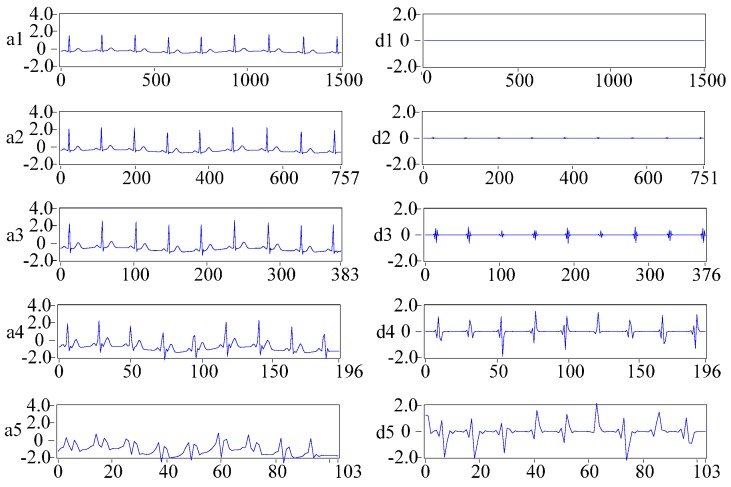
Results of wavelet transform (WT) for ECG denoising. (**a1**–**a5**) present the approximation coefficients of WT, and (**d1**–**d5**) present the detail coefficients of WT.

ECG signals are weak and often contain noise and interference. Therefore, pre-processing ECG signals before feature extraction and classification by the proposed ECG recognition system is necessary. Given that the energy distributions of ECG and noise are different, we propose an improved threshold method based on wavelet transform for denoising. The improved threshold function of the proposed denoising method is described as follows:

(1) $${\hat{w}}_{j,k}=\{\begin{array}{ll} uw+(1-u)sign(w_{j,k})\left(\left|w_{j,k}\right|-(1-b)\lambda \right) & ,\left|w_{j,k}\right|\geq \lambda \\ \;b\;sign(w_{j,k})\left(\frac{{w_{j,k}}^{4}}{\lambda^{3}}\right) & ,\left|w_{j,k}\right|<\lambda \end{array},$$

where $u=1-1/e^{\left(a\cdot {\left(\left|w_{j,k}\right|-\lambda \right)}^{2}\right)}$, *w_j,k_* is the wavelet coefficient, ${\hat{w}}_{j,k}$ is the wavelet coefficient obtained by wavelet threshold processing, *λ* is the critical threshold, *a* and *b* are regulatory factors and *a* can be any positive integer, 0≤b≤0.1. By adjusting the values of *a* and *b*, the continuity of the threshold function is achieved at the critical point, and the attenuation of the reconstructed signal is reduced, thus overcoming the inadequacy of traditional threshold functions. The critical threshold is $\lambda =\sigma \sqrt{2\log N}$, and the noise intensity is $\sigma =(mediam\left|w_{j,k}\right|)/0.6745$ in the proposed ECG denoising method [35]. We choose sym6 as the mother wavelet and utilise WT based on the Mallat algorithm [36] to decompose ECG signals into five levels, and the decomposition results are shown in Figure 2. The wavelet coefficients are then quantized with the improved wavelet threshold method to remove noise wavelet coefficients. The resulting new wavelet coefficients are utilised to reconstruct ECG signals on the basis of the Mallat algorithm to obtain the denoised ECG signals.

**Figure 3:**
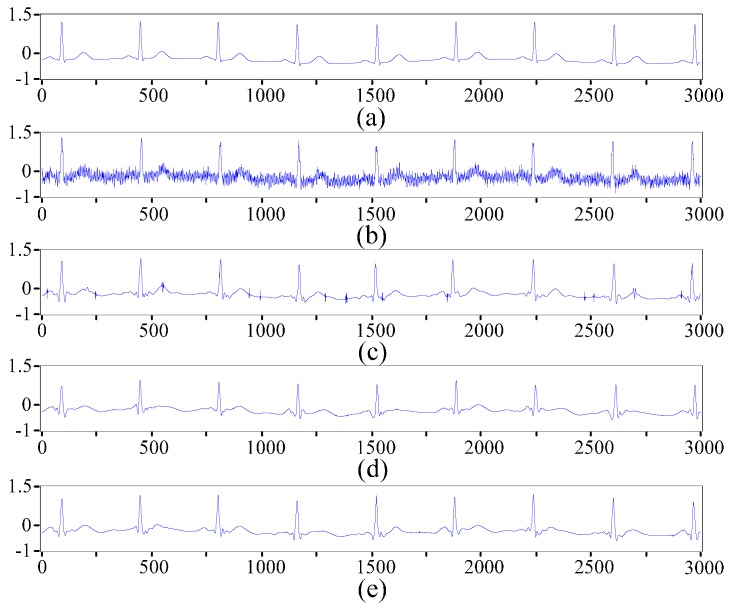
Denoising results of different threshold functions. (**a**) Original signal; (**b**) Noisy signal; (**c**) Signal denoised by the soft threshold function; (**d**) Signal denoised by the hard threshold function; and (**e**) Signal denoised by the improved threshold function.

**Figure 4:**
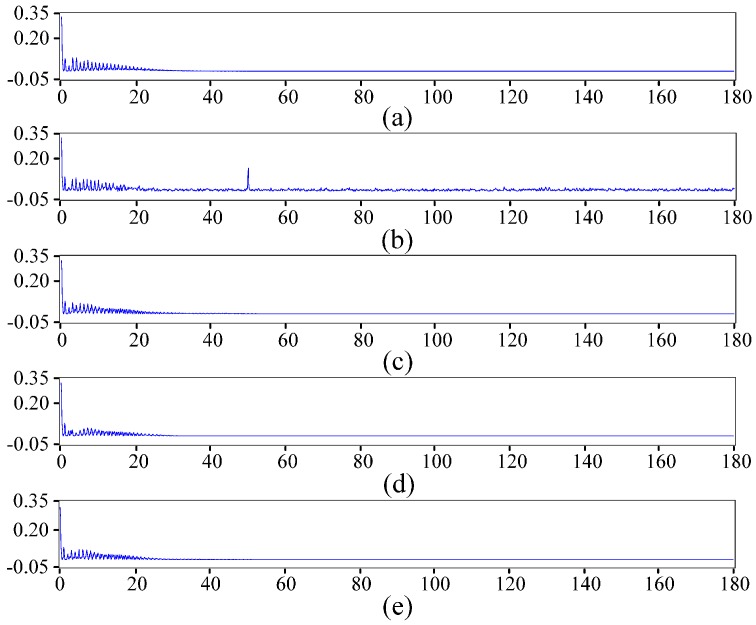
Spectrum of different signals. (**a**) Spectrum of the original signal; (**b**) Spectrum of the noisy signal; (**c**) Spectrum of the signal denoised by the soft threshold function; (**d**) Spectrum of the signal denoised by the hard threshold function; (**e**) Spectrum of the signal denoised by the improved threshold function.

To verify the utility of the presented method in ECG denoising, we obtain a noisy signal for processing with the proposed method by adding white noise as electromyographical interference and 50 Hz power-line interference to a 3000 point normal signal from the MIT-BIH arrhythmia database. The noisy signal is also processed by the hard threshold and soft threshold methods for comparison with the proposed denoising method. Figure 3 and Figure 4 show the denoising results of different threshold functions and the spectra of different signals. For better evaluating the performance of different denoising methods, signal-noise ratio (*SNR*) and root mean square error (*RMSE*) are computed based on the following equations:

(2) $$SNR=10\log\frac{\sum_{i=1}^{n}x^{2}(t)}{\sum_{i=1}^{n}{\left(x(t)-\hat{x}(t)\right)}^{2}},$$

(3) $$RMSE=\sqrt{\frac{1}{n}\sum_{i=1}^{n}{\left(x(t)-\hat{x}(t)\right)}^{2}},$$

where *x*(*t*) is the original signal, $\hat{x}(t)$ is the denoised signal and *n* is the sampling length. While the *SNR* is bigger and *RMSE* is smaller, the denoised effect is better. Five ECG signal records from MIT-BIH arrhythmia database are selected to be processed by hard threshold, soft threshold and improved threshold, respectively. The performance indicators of three threshold denoising methods are shown in Table 1, which indicates the proposed denoising method based on the improved wavelet threshold performs well relatively.

### 2.3. Multi-Domain Feature Extraction

#### 2.3.1. KICA for the Nonlinear Feature Extraction

ECG is mixed with a variety of information, and most of the important information is often included in the nonlinear process [37]. Thus, nonlinear feature extraction helps achieve better recognition of ECG beats. The method of feature extraction based on KICA is presented to extract nonlinear features in the proposed ECG recognition system. Given that high-dimensional data result in a large amount of calculation, we apply PCA [38] to reduce ECG data dimensions prior to nonlinear feature extraction by KICA. The PCA algorithm computes the eigenvalues *λ_i_* (*i* = 1, 2, …, *k*) and eigenvectors *p* of the covariance matrix *C* of ECG samples. Here, *k* is the rank of the covariance. The contribution and cumulative contribution rates are calculated according to the size of eigenvalue *λ_i_* sequenced in decreasing order. This study selects 99.8% of the cumulative contribution rate. We filter the corresponding eigenvectors of the first 20 maximum eigenvalues (*λ*_1_, *λ*_2_, …, *λ*_20_) of covariance matrix *C*. A total of 20 dimensions from the original data are obtained as the input data of KICA.

KICA [31] is a kernel method introduced in the ICA algorithm to solve the classification problem of complex nonlinear structures. KICA is applied in reconstruction within kernel Hilbert space (RKHS) nonlinear functions as comparison functions to obtain signals from a low-dimensional space mapped to a high-dimensional space. The radial basis function (RBF) kernel is selected as the kernel function of KICA to realize nonlinear transformation in this study and is defined below:

(4) $$K\left(x_{i},x\right)=\exp(\frac{-{\left\|x_{i}-x\right\|}^{2}}{2\sigma^{2}}),$$

where *δ* is a positive real number.

The concrete steps of KICA used in nonlinear feature extraction are as follows:

1. Enter ECG data and determine the number of sources *p*.
2. Initialize the decomposition matrix *W*.
3. Evaluate the source signals, *s_i_ = Wx_i_*, and compute the centralized Gram matrix, *K_1_, …, K_p_*. Here, *K_i_* = *k*(*x_i_*,*x*) (*I* = 1, 2, …, *p*).
4. Compute the minimum eigenvalue, *λ_M_ (K*_1_, …, *K_p_*), of the generalized eigenvector equation, *Kα = λDα*.
5. Compute the target function:
  (5) $$C\left(W\right)=-0.5\log_{2}\lambda_{M}\left(K_{1},\;\ldots ,\;K_{p}\right).$$
6. Minimize the target function *C(W)* and output *W*.

**Figure 5:**
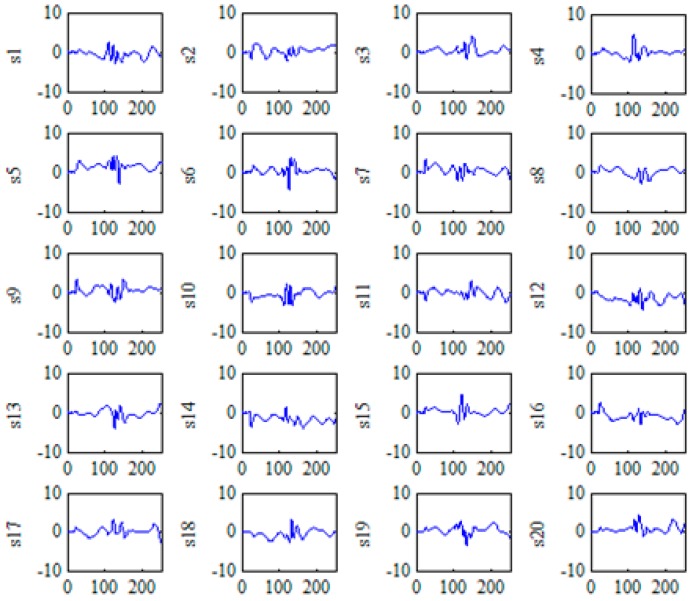
Feature subspace obtained through kernel-independent component analysis (KICA). (**s1**–**s20**) are 20 independent base signals, *x*-axis represents sample points of the ECG signal and *y*-axis is the amplitude.

After *W* is attained by KICA decomposition, feature subspace *S* is constructed by the set of independent components (*s_i_* = *Wx_i_*). The coefficient of each ECG beat mapped to the feature subspace is obtained as the nonlinear feature vector. The pseudo inverse method is applied to recognize the projection coefficient vector based on the following equation:

(6) $$A_{i}=x_{i}\;\times \;S^{-1},$$

where *A_i_* is the nonlinear feature vector of the recognized ECG beats and *S*^−1^ is the pseudo inverse matrix of independent signal vector *S*. We set the regularization parameter *kap* = 0.02 and the nuclear width of RBF kernel function *δ* = 1 in the KICA decomposition. The feature subspace obtained by using KICA is shown in Figure 5.

#### 2.3.2. DWT for Frequency Domain Feature Extraction

**Figure 6:**
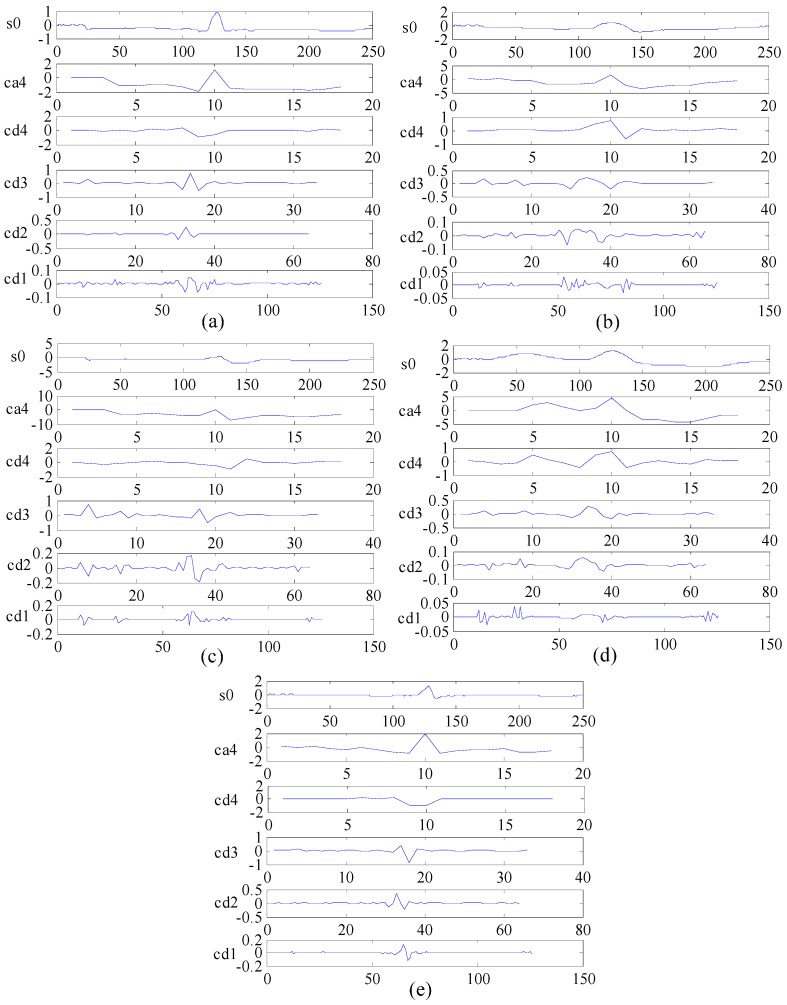
Frequency domain features of five types of ECG beats obtained through DWT. **s0** is the original ECG beat, **ca4** is the approximation of the 4th level and cd1 to **cd4** are the details of each level. (**a**) ECG of record 100 is used to represent N; (**b**) ECG of record 109 represents LBBB; (**c**) ECG of record 118 represents RBBB; (**d**) ECG of record 106 represents PVC; and (**e**) ECG of record 209 represents APC.

The DWT method based on multi-resolution analysis possesses good properties concerning time-frequency localization and is widely used in biomedical signal processing and analysis [22,23,24]. This study employs DWT to extract the frequency domain features of ECG beats in the proposed system. Daubechies 2 (db2) is selected as the mother wavelet because of its good smoothing effect [39]. The ECG beats are broken down into four levels, and the detail coefficients of each level and the approximation coefficient of the 4th level are set as ECG frequency domain features. We select records 100, 109, 118, 106 and 209 from the MIT-BIH arrhythmia database to represent five types of ECG beats, namely, N, LBBB, RBBB, PVC and APC, for decomposition with DWT as shown in Figure 6. The maximum, minimum, mean and standard deviation values of the wavelet coefficients of the ECG signal are calculated as new frequency domain features. We also utilise LDA [32] as a dimension reduction tool to extract more effective frequency domain features in this study.

### 2.4. Classification Based on SVM Optimized by GA

SVM, which was developed by Vapnik [40], has been widely applied in ECG classification studies [23,25,28,29]. SVM finds a hyperplane in a high-dimensional space by separating the training samples of each class or by maximizing the minimum distance between the hyperplane and training samples. Our proposed ECG recognition system utilises a library for SVM (LIBSVM) [41] as the classifier and adopts the popular RBF function as a kernel function. To improve the performance of the classifier, GA, a type of optimization searching algorithm based on the Darwinian principle of survival of the fittest [42,43], is also employed to optimize the parameters *C* and *δ* of the RBF kernel function. The chromosome of GA contains two genes because the chromosome of SVM contains separate values for kernel parameters *δ* and *C*. The initial parameters of GA are max iteration = 200, max number of population = 20 and parameter of cross validation = 5. The value range of penalty factor *C* is 0–100. The value range of RBF kernel width *δ* is 0–1000. The basic parameter optimization of SVM with GA is described below:

1. Generate an initial population with the binary code.
2. Compute the fitness function and the training set for CV to determine the fitness function value.
3. Filter a new population from the old population on the basis of individual fitness, which is determined by the evaluation function.
4. Employ several genetic operators of mutation and crossover to generate new solutions.
5. Calculate the fitness function of the newly generated individuals.
6. Repeat steps 3–5 until the maximum iteration is reached and then find the optimal parameters.

## 3. Experimental Design

**Figure 7:**
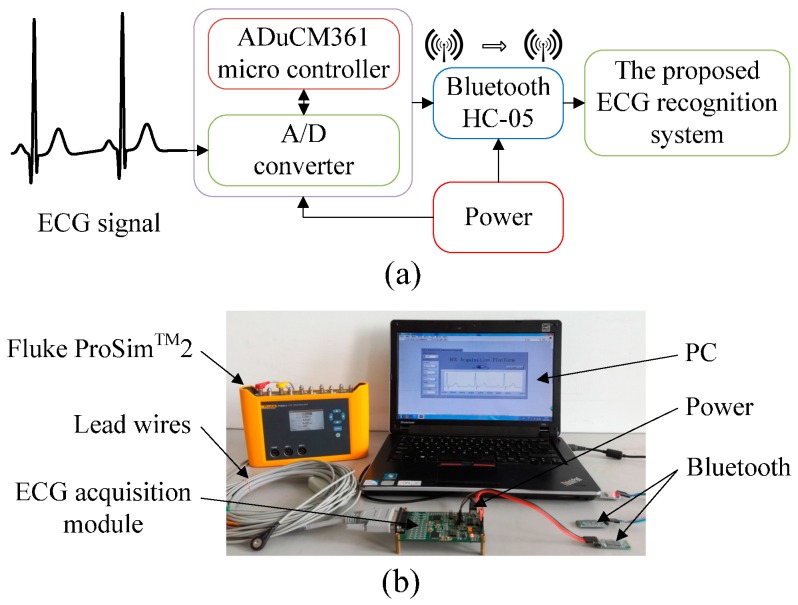
Diagrams of the ECG acquisition experimental platform. (**a**) Schematic of the experimental platform; and (**b**) Construction of the experimental platform.

We construct an ECG acquisition experimental platform to collect ECG data for arrhythmia recognition to verify the validity of the proposed system. Fluke ProSim^TM^2 vital sign simulator is used as the signal resource to provide different types of ECG signals, and standard I lead ECG is selected in this platform. An ECG acquisition module is a critical part of the experimental platform and is integrated with an ADuCM361 (Analog Devices, Inc., Norwood, MA, USA) micro controller and an A/D converter (Analog Devices, Inc., Norwood, MA, USA) to acquire analog ECG signals and convert them into digital ones. The collected ECG data are transmitted via Bluetooth HC-05 (DX-Smart Technology Co. Ltd., Shenzhen, China) to a PC, and the data are then input into the proposed ECG recognition system for classification. The power module is used to supply electricity to the ECG acquisition module and the Bluetooth module. Figure 7 provides diagrams of the ECG acquisition experimental platform.

## 4. Results and Discussion

### 4.1. Results of the Proposed ECG Recognition System

#### 4.1.1. Multi-Domain Feature Extraction Using KICA Combined with DWT

A novel method based on multi-domain feature extraction is proposed to extract many effective features for ECG recognition. ECG data from the MIT-BIH arrhythmia database are sampled, and the data are pre-processed with the improved wavelet threshold method. Given that the amplitude of the R wave is the most outstanding in the ECG signal, the R wave position serves as the benchmark before and after sampling 250 time domain points containing the complete QRS complex of the ECG signal. A total of 1800 samples from the MIT-BIH arrhythmia database are equally divided into training sets. A total of 400 samples of N are derived from records 100, 101, 103 and 105. Similarly, 400 samples of LBBB are derived from records 109, 111, 207 and 214, and 400 samples of RBBB are derived from records 118, 124, 212 and 231. We also derive 400 samples of PVC from records 106, 119, 200 and 203 and 200 samples of APC from records 209 and 222. A total of 1800 samples are used as ECG data after sampling and pre-processing the ECG signals. Each sample contains 250 points for multi-domain feature extraction. A matrix of 1800 × 250 with high dimensions requires extensive calculation for KICA. To obtain effective features from the ECG signals, we reduce the dimensions of ECG data through PCA. The method of PCA dimension reduction is used, and the cumulative contribution rate is set to 99.8%. Twenty dimensions are selected from the original set as KICA input. Thereafter, KICA is applied to extract the nonlinear features of five types of ECG beats for classification with the proposed system. In the algorithm of nonlinear feature extraction using KICA, the regularization parameter is *kap* = 0.02, and the nuclear width of the RBF kernel function is *δ* = 1. A set of statistically independent base signals, *s_i_* (*i* = 1, …, 20), is obtained, and the feature subspace is constructed with the base signal vector. The coefficient of each projection to the feature subspace is for nonlinear features. Therefore, a feature matrix of 1800 × 20 is obtained as the nonlinear features after KICA.

This study applies DWT to extract the frequency domain features of ECG beats, and db2 is selected as the mother wavelet function to decompose ECG beats into four levels. The details of each level and the approximation of the 4th level as the frequency domain features are computed to obtain the statistical features, which include the maximum, minimum, mean and standard deviation values, of each wavelet coefficient. After 20 dimensions of the frequency domain features are obtained by DWT combined with the statistical method, LDA is utilised to optimise these features reduced to four dimensions. Therefore, a feature vector with a 1800 × 4 matrix is derived as the frequency domain features.

#### 4.1.2. Classification Using SVM Optimized by GA

**Figure 8:**
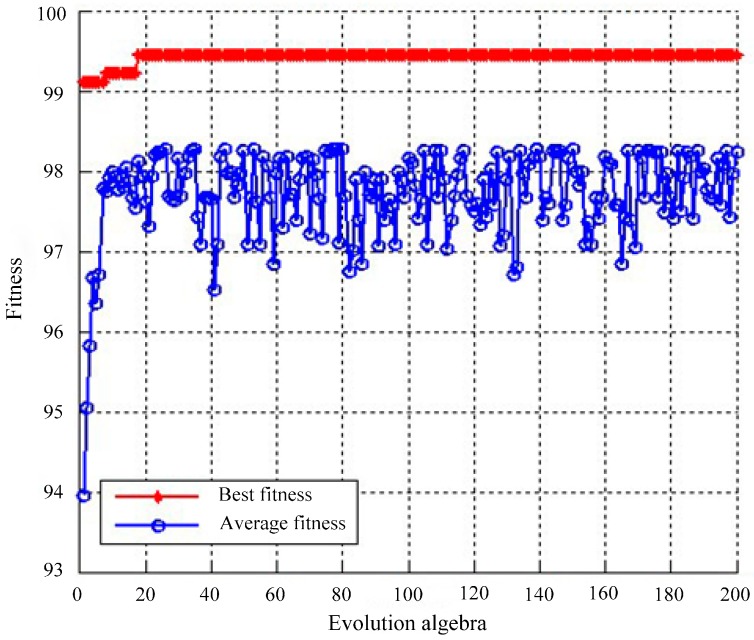
Fitness curve of GA for finding the optimal parameters of SVM. Average fitness and best fitness are gradually increased via a series of iterations. When the evolution algebra is 200, average fitness and best fitness reach the maximum value, namely, the final optimization parameters of SVM are obtained.

We obtain a multi-domain feature vector with a 1800 × 24 matrix by KICA combined with DWT. The matrix is used as an input to train and test the SVM classifier. ECG beats are identified by LIBSVM, and RBF is applied to classify multi-domain features. The selection of the kernel function and parameters is extremely important for SVM. The selection of penalty factor *C* and RBF kernel width *δ* determines the classifier’s performance, and the optimal parameters can effectively prevent overlearning and lack of a learning state. Therefore, we employ GA to determine the optimal parameters of SVM in the proposed system. The final optimization results show that *C* = 2.61633 and *δ* = 4.16832. The fitness curve of GA for finding the optimal parameters of SVM is shown in Figure 8.

Accuracy (*A_CC_*), sensitivity (*Se*), specificity (*Sp*) and positive predictivity (*Pp*) are evaluated to investigate the recognition performance of the proposed system. The performance parameters for each class are defined as follows:

(7) $$A_{CC}=\frac{TP+TN}{TP+TN+FP+FN}\times 100\%=\frac{N_{T}-N_{E}}{N_{T}}\times 100\%$$

(8) $$S_{e}=\frac{TP}{TP+FN}\times 100\%$$

(9) $$S_{p}=\frac{TN}{TN+FP}\times 100\%$$

(10) $$P_{p}=\frac{TP}{TP+FP}\times 100\%$$

where *TP* is true positive, *FP* is false positive, *FN* is false negative and *TN* is true negative. *N_E_* and *N_T_* represent the total number of classification errors and the total number of the testing sets, respectively. Table 2 and Table 3 present the multi-domain feature classification results obtained with the SVM classifier. Table 2 shows that the five types of ECG beats produce different classification results. N and RBBB samples are correctly classified. Two LBBB samples are incorrectly classified as PVC. Five PVC samples are classified as LBBB. Three APC samples are incorrectly classified as N, and one sample is classified as PVC. Table 3 clearly shows that the five types of ECG beats perform well in classification. RBBB has the best statistical performance indicators of sensitivity, specificity and positive predictive value amongst the five types of ECG beats. APC presents good performance with a specificity of 100% and a positive predictive value of 100%; its sensitivity of 96% is the lowest among the sensitivities of other types. N, LBBB and PVC present similar statistical performance indicators.

### 4.2. Experimental Results from the ECG Acquisition Platform

**Figure 9:**
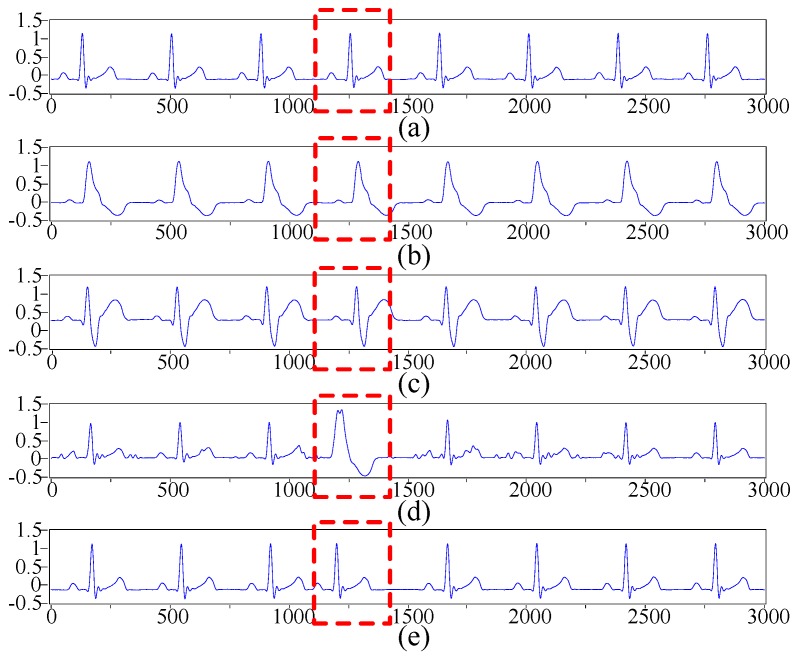
Five types of ECG signals acquired by the experimental platform. The ECG signals in the red dashed boxes are the five types of the acquired beats. (**a**) N; (**b**) LBBB; (**c**) RBBB; (**d**) PVC; and (**e**) APC.

The five types of ECG signals acquired by the designed experimental platform (see Figure 9) are entered into the proposed ECG recognition system to be classified. Firstly, noise is removed from the collected ECG signals with the improved wavelet threshold method. Secondly, 1800 ECG beats are sampled and utilised to obtain the nonlinear and frequency domain features of ECG beats with the proposed method. Thirdly, PCA is employed to reduce the dimensions of ECG sample data in the nonlinear feature extraction, and LDA is applied to reduce the dimensions of the frequency domain features. The multi-domain features of the ECG beats are then classified with LIBSVM, and GA is adopted to optimise the penalty factor and the kernel width of the classifier. Lastly, we utilise the proposed ECG recognition system to perform experimental classification. The classification results are shown in Table 4 and Table 5. Table 4 shows that the five types of ECG beats produced different classification results. Two N samples are incorrectly classified as LBBB, and one N sample is classified as PVC. Four LBBB samples are incorrectly classified as N. Two samples of LBBB are classified as PVC. Only one sample of RBBB is incorrectly classified as PVC. Eleven PVC samples are incorrectly classified. One sample of APC is incorrectly classified as N. Table 5 shows that the classification of the five types of ECG beats achieves an overall accuracy of 97.3%. RBBB and APC present the best statistical performance indicators of sensitivity, specificity and positive predictive value amongst the five types of ECG beats. PVC has a good performance with a specificity of 99.43% and a positive predictive value of 97.93%; its sensitivity of 94.50% is the lowest amongst the sensitivities of the other types. N and LBBB possess similar statistical performance indicators.

### 4.3. Discussion and Comparisons

Table 3 and Table 5 clearly show that the proposed ECG recognition system achieves satisfactory identification results for two types of ECG data. The proposed system using the MIT-BIH arrhythmia database presents excellent performance with an accuracy of 98.80%, average sensitivity of 98.50%, average specificity of 99.69% and average positive specificity of 98.91%. Experimental classification results from the ECG acquisition experimental platform show that the accuracy, average sensitivity, average specificity and average positive specificity are 97.30%, 97.50%, 99.32% and 97.41%, respectively. Although the classification results using ECG data from the ECG acquisition experimental platform are good, the proposed system using ECG data from the MIT-BIH arrhythmia database achieves better results and classification accuracy based on the three statistical performance indicators. The primary reason is that ECG signals from Fluke ProSim^TM^2 are limited in providing a large number of ECG samples. Another important reason is that several types of ECG signals might require other features to represent their characteristic information. Therefore, further study and evaluation of the algorithm based on feature extraction and classification in the ECG recognition system are necessary.

The results of the proposed ECG recognition system are compared with those of other reported ECG classification methods. The comparison results are shown in Table 6. Martis utilised morphological and time features and used LSSVM to classify five ECG classes (N, RBBB, LBBB, APC and PVC); accuracy of 93.48% was achieved [27]. Kamath derived ECG features based on Teager energy functions, classified five types of beats (N, PVC, Paced beat, RBBB and LBBB) with an NN classifier and obtained a recognition accuracy of 95% [30]. In another study, morphological and time features as effective features for three beat classes (N, PVC and other beats) were used as input for an SVM classifier with a classification accuracy of 97.14% [44]. The SVM classifier was used to identify four types of ECG (N, atrial arrhythmia, conduction block and ventricular arrhythmia) based on the time interval feature extracted via biorthogonal spline wavelet, which achieved an accuracy of 95.65% [20]. Liu presented an algorithm based on SVM to detect and mark ECG signals. The algorithm utilised the self-constructing neural fuzzy interference network (SoNFIN) to recognize five ECG signal types (N, PVC, PAC, LBBB and RBBB) with an accuracy of 96.4% [21]. As shown in Table 6, the proposed ECG recognition system based on multi-domain feature extraction using KICA combined with DWT achieves higher accuracy of 98.8% in comparison with other methods.

## 5. Conclusions

A novel ECG recognition system based on multi-domain feature extraction using KICA and combined with DWT is proposed to classify five types of ECG heartbeats. A new improved threshold wavelet method for ECG pre-processing is also presented to eliminate the influence of noise. In multi-domain feature extraction, PCA as a feature reduction method is employed to reduce ECG data dimensions and decrease calculation in KICA nonlinear feature extraction. LDA is also adopted to optimise the frequency domain features extracted by DWT. SVM is utilised as the classifier to recognise ECG beats. We apply GA to improve the performance of the SVM classifier by optimizing RBF kernel function parameters *δ* and *C*. The proposed system with the MIT-BIH arrhythmia database for ECG beat classification achieves 98.8% accuracy, 98.50% sensitivity, 99.69% specificity and 98.91% positive predictability. We also construct an ECG acquisition experimental platform for ECG data collection to verify the effectiveness of the proposed system. The results of the experimental ECG acquisition platform show that the system obtains excellent results with 97.3% accuracy, 97.5% sensitivity, 99.32% specificity and 97.41% positive predictability. The presented system is able to achieve satisfactory classification results in classifying five types of ECG beats. Therefore, the proposed ECG recognition system can be used to effectively diagnose heart diseases.

## Figures and Tables

**Table 1:** Performance indicators of three threshold denoising method.

Methods	Performance Indicators	ECG Signal Records				
		103	102	118	232	231
Hard threshold	signal-noise ratio (*SNR*)	21.2769	20.4320	21.8957	18.1098	22.6279
	root mean square error (*RMSE*)	0.0255	0.0252	0.0257	0.0319	0.0221
Soft threshold	*SNR*	21.7454	20.2268	22.1032	18.0057	22.5574
	*RMSE*	0.0241	0.0258	0.0251	0.0328	0.0223
Improved threshold	*SNR*	24.0626	23.4869	24.0128	20.8680	25.2851
	*RMSE*	0.0185	0.0178	0.0179	0.0236	0.0163

**Table 2:** Classification results of the support vector machine (SVM) classifier.

Type	N	LBBB	RBBB	PVC	APC
normal beat (N)	200	0	0	0	0
left bundle branch block beat (LBBB)	0	198	0	2	0
right bundle branch block beat (RBBB)	0	0	200	0	0
premature ventricular contraction (PVC)	0	5	0	195	0
atrial premature beat (APC)	3	0	0	1	96

**Table 3:** Statistical performance indicators of the SVM classifier: sensitivity (*Se*), specificity (*Sp*), and positive predictability (*Pp*).

Type	Sensitivity (*Se*)	Specificity (*Sp*)	Positive Predictability (*Pp*)
normal beat (N)	100%	99.57%	98.52%
LBBB	99%	99.29%	97.54%
RBBB	100%	100%	100%
PVC	97.50%	99.57%	98.48%
APC	96%	100%	100%
Average	98.50%	99.69%	98.91%
Accuracy(*Acc*)		98.80%	

**Table 4:** The classification results based on electrocardiogram (ECG) acquisition experiment platform.

Type	N	LBBB	RBBB	PVC	APC
N	195	2	0	1	2
LBBB	4	194	0	2	0
RBBB	0	0	199	1	0
PVC	1	8	2	189	0
APC	1	0	0	0	99

**Table 5:** The performance statistical indicators of the experiment results: sensitivity (*Se*) and specificity (*Sp*), and positive predictability (*Pp*).

Type	*Se*	*Sp*	*Pp*
N	97.5%	99.14%	97.01%
LBBB	97%	98.57%	95.10%
RBBB	99.50%	99.71%	99%
PVC	94.50%	99.43%	97.93%
APC	99%	99.75%	98.02%
Average	97.50%	99.32%	97.41%
*Acc*		97.30%	

**Table 6:** Comparison results of the proposed system with other literatures.

Methods	Classifier	Classes	Accuracy	Reference
Principal components of bispectrum features	least squares support vector machine (LSSVM)	5	93.48%	Martis et al.
Teager energy function features	neural network (NN)	5	95%	Kamath
Morphological and time features	support vector machine (SVM)	3	97.14%	Zadeh et al.
Time intervals	SVM	5	95.65%	Fei
R-R intervals	self-constructing neural fuzzy interference network (SoNFIN)	5	96.4%	Liu
The multi-domain features	a library for SVM (LIBSVM)	5	98.8%	Proposed method
